# Perspectives of Persons With Dementia, Family, and Healthcare Providers on an Advance Care Planning Decision Aid

**DOI:** 10.1093/geroni/igaa057.2849

**Published:** 2020-12-16

**Authors:** Lisa Geshell, Jung Kwak

**Affiliations:** 1 The University of Texas at Austin, Austin, Texas, United States; 2 University of Texas at Austin, Austin, Texas, United States

## Abstract

Few evidence-based interventions to engage persons living with dementia in advance care planning (ACP) exist. We developed and explored the acceptability and appropriateness of an ACP decision aid for PLWD/family caregiver dyads living in the U.S. We conducted a mixed-methods study with 10 persons with dementia-caregiver dyads, and 4 healthcare providers. Content analysis and descriptive analysis were conducted. Major and subthemes included: (1) the role of caregivers during ACP discussion: clarifier, guide, and proxy; (2) decisional needs of persons with dementia for ACP: lack of knowledge and decisional conflict; and (3) perceptions of the decision aid: lack of clarity between advance directive types, a need to remove any questions that may be conceptually different, but appear similar to users, and need to include summary of decision-support needs. Findings provide implications for how healthcare providers can use a decision aid to facilitate ACP conversations with persons with dementia and caregivers.

